# Effects of flowering phenology and synchrony on the reproductive success of a long-flowering shrub

**DOI:** 10.1093/aobpla/plw007

**Published:** 2016-02-02

**Authors:** Javier Rodríguez-Pérez, Anna Traveset

**Affiliations:** 1 Institut Mediterrani d'Estudis Avançats – IMEDEA (CSIC-UIB), Miquel Marqués, 21, E07190 Esporles, Mallorca, Balearic Islands, Spain; 2 Present address: Aranzadi Sciences Society, Zorroagagaina 11, E20014 Donostia-San Sebastián, Spain

**Keywords:** Balearic Islands, flower synchrony, flower visitors, *Hypericum balearicum*, Mediterranean plants, rainfall effect on reproduction, resource limitation

## Abstract

Flowering phenology and synchrony with biotic and abiotic resources are crucial traits determining the reproductive success in insect-pollinated plants. In seasonal climates, plants flowering for long periods should assure reproductive success when resources are more predictable. In this work, we evaluated the relationship between flowering phenology and synchrony and reproductive success in *Hypericum balearicum*, a shrub flowering all year round but mainly during spring and summer. We studied two contrasting localities (differing mostly in rainfall) during 3 years, and at different biological scales spanning from localities to individual flowers and fruits. We first monitored (monthly) flowering phenology and reproductive success (fruit and seed set) of plants, and assessed whether in the locality with higher rainfall plants had longer flowering phenology and synchrony and relatively higher reproductive success within or outside the flowering peak. Secondly, we censused pollinators on *H. balearicum* individuals and measured reproductive success along the flowering peak of each locality to test for an association between (i) richness and abundance of pollinators and (ii) fruit and seed set, and seed weight. We found that most flowers (∼90 %) and the highest fruit set (∼70 %) were produced during the flowering peak of each locality. Contrary to expectations, plants in the locality with lower rainfall showed more relaxed flowering phenology and synchrony and set more fruits outside the flowering peak. During the flowering peak of each locality, the reproductive success of early-flowering individuals depended on a combination of both pollinator richness and abundance and rainfall; by contrast, reproductive success of late-flowering individuals was most dependent on rainfall. Plant species flowering for long periods in seasonal climates, thus, appear to be ideal organisms to understand how flowering phenology and synchrony match with biotic and abiotic resources, and how this ultimately influences plant reproductive success.

## Abstract (precis)

Plants that flower for long periods are ideal organisms in which to test whether the timing and duration of flowering adjust to the seasonal timing of biotic and abiotic resources, and whether that influences their reproductive success. *Hypericum balearicum* is an evergreen endemic shrub that flowers all year round but mainly during spring and summer. We found that those plants inhabiting a locality with lower rainfall had the capacity to flower longer. During spring and summer, the reproduction of plants that flowered earlier depended on both pollinators and rainfall, whereas the reproduction of plants that flowered later depended more upon rainfall scarcity.

## Introduction

Flowering phenology, or the period of time when plant species flower, determines the season when reproductive structures interact with resources for fruit and seed development (Primack 1985; Rathcke and Lacey 1985). In seasonal climates, insect-pollinated plants flower during short periods, and such periods generally match with the most favourable season for pollinator activity (Elzinga *et al.* 2007). Despite flowering phenology is under genetic control, biotic and abiotic factors are also important forces triggering the expression of flowering phenology (Nicotra *et al*. 2010). If environmental conditions remained uniform and predictable, species could actually flower longer (Rathcke and Lacey 1985; Marquis 1988), thus showing weak selection on flowering phenology (Mungía-Rosas *et al.* 2011). In seasonal climates, by contrast, extended flowering is likely to assure plant reproduction, adjust plant investments in flowers and fruits, and/or avoid pre-dispersal seed predators (Bawa et al. 2003). The context dependence of abiotic and biotic conditions imposes inconsistent trends on flowering phenology and synchrony in plant populations (Parra-Tabla and Vargas 2007), making phenological traits difficult to predict (Mungía-Rosas *et al.* 2011). Within populations, plant species can extend flowering owing to (i) a plastic flowering phenology of individuals with low flowering synchrony (e.g. Tarayre *et al.* 2007) or (ii) a high synchrony of individual plants flowering for long periods (e.g. Picó and Retana 2000). Hence, it is necessary to decompose the relative effects of biotic and abiotic conditions on flowering traits, with the aim to improve our understanding of the patterns of phenotypic selection on flowering phenology (Mungía-Rosas *et al.* 2011).

In insect-pollinated plants, flowering phenology and synchrony among individuals is usually a crucial trait determining plant reproduction (Ollerton *et al.* 2011). A high floral synchrony produces a large floral display and promotes a high flower visitation rate by insects, which eventually cascades into high reproductive success (Forsyth 2003; Kudo and Harder 2005). In plant species with long-flowering phenology, flowers that develop outside the flowering peak of each locality may have lower chances to be pollinated by insects and, thus, may set less fruits and/or seeds than those developed when most flowers are receptive (e.g. Elberling 2001; Forrest and Thomson 2011). Estimates of plant reproduction usually covary with biotic and abiotic factors, especially in climates differing in the period of available plant resources. The Mediterranean climate imposes constrains on plant reproduction, as plants flowering early in the season have low chances to be insect pollinated and to produce fruits and seeds (e.g. Traveset 1995; Picó and Retana 2000; Sánchez *et al.* 2012), whereas drought reduces resources for fruit and seed development in plants flowering late in the dry season (Herrera 1992; Giménez-Benavides *et al.* 2007). Flowering phenology further affects seed mass and/or seed viability, as aridity is stronger in Mediterranean climates and influences resource allocation to fruits and/or seeds (e.g. Cavers and Steel 1984; Vaughton and Ramsey 2001). In insect-pollinated plants, it is thus crucial to study phenological consistencies and uncouplings of abiotic and biotic factors and to evaluate how these covary on each context (i.e. onset of flowering, localities) to better understand the importance of flowering phenology on reproductive success (Kudo 2006; Elzinga *et al.* 2007).

**Figure 1.**
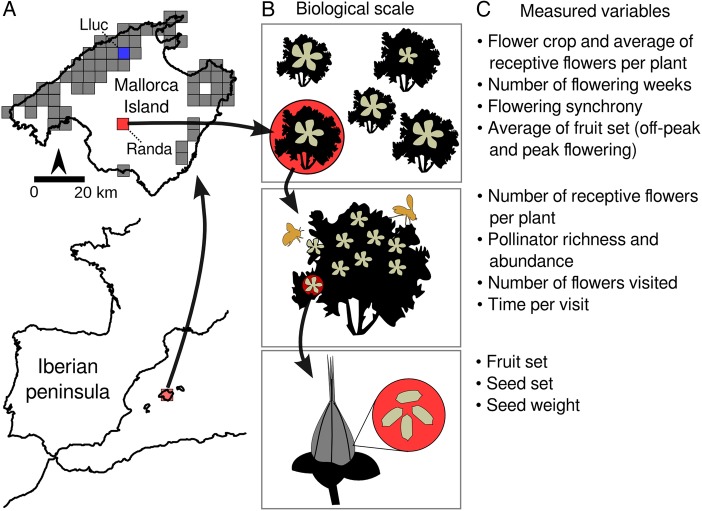
Location of study localities (left panels) and the biological scales to measure reproductive success in *H. balearicum*. (A) Grey areas depict the presence of *H. balearicum* in Mallorca Island within 5 × 5 km (Source: Bioatles 2.1; Government of the Balearic Islands), whereas red (for Randa) and blue areas (for Lluc) represent the location of studied localities. (B) Biological scales measured in our study, spanning from locality (upper panel), individual plants (mid panel) and flower and fruit (lower panel); each measured biological scale (i.e. individual plant, flower and fruit and seed) is represented by red circles. (C) Measured variables for each biological scale.

*Hypericum balearicum* (Hypericaceae) is an evergreen shrub endemic to the Balearic Islands (West Mediterranean). It flowers throughout the year, though mainly during spring and summer (Tébar *et al.* 2004), the dry season in the Mediterranean basin but with high abundance of flower visitors. Additionally, it also flowers during autumn and winter, which is the rainy season in the Mediterranean basin. This species is, thus, ideal to compare the reproductive success of flowers produced under contrasting rainfall conditions, and assess whether the species performs proportionally better in autumn–winter, when abiotic conditions are less severe. Here, we studied two localities in Mallorca Island at different biological scales spanning from locality to the individual flower and fruit (Fig. 1). We first assessed at the locality scale whether flowering phenology and synchrony, flower crop and number of receptive flowers varied between the period when most flowers are receptive (flowering peak) than outside this period (flowering off-peak), and how this affects the reproductive success of *H. balearicum* individual plants. We hypothesized that plants under favourable abiotic conditions would have more relaxed flowering phenology and synchrony, and equivalent reproductive success outside this period (due to lower competition for pollinators). Secondly, we tested if reproductive success (i.e. fruit and seed set and seed weight) of individuals during the flowering peak of each locality was associated with richness, abundance and visitation rates of flower visitors. If that association was positive, we could further predict that pollinators limit the reproductive success during the flowering peak; otherwise, water availability might likely limit reproductive success, especially at the end of the summer period when rainfall is scarce in the Mediterranean basin.

## Methods

### Study species

*Hypericum balearicum* (Hypericaceae) is a perennial shrub that can reach up to 1.5 m. This species is endemic to the Balearic Islands, inhabits four of the five main islands, and it has naturalized in the Ligurian region (South Italian peninsula; Ramos-Núñez 2001). *Hypericum balearicum* is closely related to *H. calycinum* (Pilepić *et al*. 2011), native to Southeastern Europe and invasive worldwide. In Mallorca Island, *H. balearicum* is abundant in the Tramuntana mountain range (West Mallorca), with small and scattered localities in the centre and northeast of the island (Fig. 1; Ramos-Núñez 2001).

*Hypericum balearicum* mainly flowers from early May to mid-July, but it frequently flowers outside this period, even in winter—although much less common (Tébar *et al.* 2004). Such extended flowering is a trait not found in the other 29 *Hypericum* species of the Iberian Peninsula (Ramos-Núñez 2001). Flowers are pentamerous, hermaphroditic, yellow and solitary (flower diameter: ∼4 cm) with numerous stamens (87 ± 2, *n* = 54), located at the tip of new branches (Fig. 1B). Flower lifespan is ∼24 h (J. Rodríguez-Pérez, pers. obs.). According to Cruden (1977), *H. balearicum* is allogamous due to the high pollen/ovule ratio (i.e. 3597; Tébar *et al.* 2004). Wind pollination is almost negligible (Tébar *et al.* 2004). Flowers of *H. balearicum* are self-compatible (<30 % flowers set fruits in spontaneous selfing experiments) and are not pollen limited when most flowers are produced (J. Rodríguez-Pérez, unpublished data). Flowers have no apparent nectaries, and thus, only pollen is offered as a reward to flower visitors (Fig. 1B). Fruits are resiniferous capsules (Fig. 1C) containing dry and tiny seeds (1.33 ± 0.02 mm; 0.25 ± 0.01 mg), without signs of any apparent dispersal syndrome.

### Study sites

The study took place in two localities at Mallorca Island (Fig. 1): Randa (31N 493172, 4374825; 350 m above sea level (a.s.l.)) and Lluc (31N 490548, 4406824; 550 m a.s.l.). Mallorca has a strong precipitation gradient from southeast to northwest, spanning from ∼300 to >1000 mm, respectively (AEMET Spanish Meteorological Agency). Randa is a small and isolated locality with a very small population of *H. balearicum* (up to 15 individuals) and with an annual rainfall of only 522 mm (AEMET Spanish Meteorological Agency). Lluc, by contrast, is a locality in the middle of the Tramuntana mountains (Fig. 1; Ramos-Núñez 2001), where the species is abundant and less isolated, and that receives twice as much rainfall (1379 mm) as Randa. The main vegetation in Randa is typical Mediterranean scrubland in which species like *Pistacia lentiscus*, *Olea europea*, *Phillyrea angustifolia*, *Cistus albidus* and *Quercus ilex* predominate. By contrast, the main vegetation in Lluc is holm oak (*Q. Ilex*) forest, mixed with *Pinus halepensis*, *Pistacia lentiscus*, *C. monspeliensis*, *Rhamnus ludovici-salvatoris* and *Cneorum tricoccon*. Plants in both localities occur in riverbeds and banks of temporal streams, and they have a similar density (1.02 and 1.14 plants ha^−1^, for Randa and Lluc, respectively).

### Flowering phenology and synchrony

**Figure 2.**
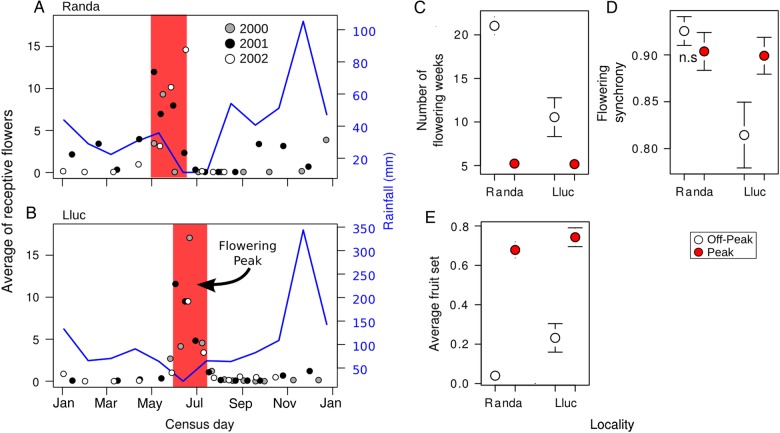
Relationship between flowering phenology, synchrony and reproductive success of *H. balearicum*. In the left panels (A and B), we plotted the average fruit crop for census days. Circles represent the average of receptive flowers per plant and census day, and years are in different colours. In each locality, census (days) inside the red area are within the flowering peak, whereas those outside of it are within the flowering off-peak. In the right panels, we plotted differences in flowering season (in colours) and locality (*x*-axes) for (C) the number of flowering weeks, (D) flowering synchrony and (E) average fruit set (i.e. fruits averaged per plant and census). In the right panels (C–E), circles represent values per plant (mean ± SE).

During May 2000, 11 plants in Randa and 15 in Lluc were tagged and monitored until October 2002. For each locality, plants were fortnightly checked during the flowering peak, and once per month throughout the rest of the year except in Lluc, where plants were monitored fortnightly up to last September due to the presence of flower buds (Fig. 2). Each locality has flowering peaks in different periods (Fig. 2A and B), and we thus delimited the ‘flowering peak’ to the period when the average number of receptive flowers per plant is greater than five in each locality and year (red area in Fig. 2A and B); we thus defined ‘flowering peak as an arbitrary threshold metric of large availability of flowers for reproduction. Conversely, we defined ‘flowering off-peak’ as the time lag outside the ‘flowering peak’ in each locality, which spans during the autumn and winter seasons (Fig. 1A and B).

At each census day and individual plant, we recorded the number of receptive flowers, and we further calculated the following variables: (i) flower crop (number of flowers produced per flowering season), (ii) number of flowering weeks (difference between the first and last periods with open flowers) and (iii) flowering synchrony, defined as the degree to which plant's flowering duration (weeks in our case) overlapped with the rest of individuals in the locality, following Augspurger (1983). The index of synchrony (*X*) for the plant *i* is given by

$$X_{i}=\left(\frac{1}{n-1}\right)\left(\frac{1}{\;f_{i}}\right)\sum_{\;j=i}^{n}e_{\;j\neq i}$$

where *e_j_* is the number of weeks that the plants *i* and *j* overlapped in the flowering, *f_i_* is the total number of weeks individual *i* was in flower and *n* is the number of plants in the sample. *X* varies from 0 (no overlap) to 1 (the flowering of a given plant overlaps with that of all plants in the locality).

### Pollinators during flowering peak

During the flowering peak of each locality, we censused flower visitors (pollinators, hereafter, regardless of their effectiveness as ‘legitimate’ pollinators, which would require a more in-depth study of their performance on reproductive output) to determine their species richness and abundance, and their rate of flower visitation. At each locality and day, we censused flowering individuals with at least one receptive flower; censuses were performed during 2 years (2001–02) in Randa and during only 1 year in Lluc (2001). We censused 12 plants per locality, including when possible the individuals used to study flowering phenology and synchrony and reproductive success. Preliminary observations during the autumn and winter seasons suggested very low flower visitation rates (0.44 visits h^−1^), and thus, systematic censuses were not performed during that period (J. Rodríguez-Pérez, unpublished data). At Randa, we censused pollinators during a total of 29.75 h, whereas in Lluc, during 23.50 h. During daytime (from 10:00 to 17:00 hours) and on warm sunny days, we haphazardly choose single flowering plants, and we performed censuses on them; we followed the same individuals at different daytimes along the flowering season. During each census, lasting 15 min, the following variables were recorded: (i) order of pollinators, and species when possible, (ii) number of visited flowers and (iii) time per visit (s), i.e. time spent on each individual plant visiting flowers. For beetles and ants, only the number of individuals per species was recorded because they can remain on one flower for long periods (>15 min). At the end of each census, we also recorded the number of receptive flowers per plant.

### Reproductive success

At different days and in each locality, we tagged a random sample of three receptive flowers per individual plant to measure both fruit and seed set; we tagged flowers on 11 plants in Randa and 15 on Lluc. As soon as fruits ripened (∼50 days after flowers were receptive), they were individually collected and taken to the laboratory to be measured. For each tagged flower, fruit set was 1 if the flower developed a fruit (with at least one seed), or 0 if that flower aborted. Each developed fruit was thus dissected to determine seed set (i.e. number of seeds relative to number of ovules). Due to the small seed size, we obtained seed weight by weighing all seeds within a fruit (to the nearest 0.1 mg) and dividing it by the total number of seeds.

### Data analysis

We used generalized linear models (GLMs) and generalized linear mixed models (GLMMs) to analyse how variables related to (a) flowering phenology and synchrony per plant (Table 1), (b) pollinators during the flowering peak of each locality (Table 2) and (c) reproductive success during the flowering peak (Table 3) were affected by predictor variables (locality, year, pollinator order and flowering season). A different set of covariates for each group of analyses was also included in the models (see details in Tables 1–3); for instance, flower crop per plant for (a) analyses (see Table 1), number of receptive flowers per plant and census day for (b) analyses (Table 2) and number of seeds per fruit for (c) analyses (Table 3). Prior to (a) analyses, we performed cross-correlations between pairs of dependent variables in order to test the collinearity between them **[see** **Supporting Information—Fig. S1****]**. For analyses related to (a), we used GLMs with individual plant at each locality and individual plant per census day as replication units; for (b), we used GLMs and individual plant as a replication unit, and GLMMs and individual pollinator visit per plant as replication unit and individual plant as random factor; finally, for analyses related to (c), we performed GLMMs with individual fruit as the replication unit, and the individual plant as random factor. We included the error distributions and link functions that best fitted each dependent variable (see details in Tables 1–3). We consistently selected the most parsimonious model among all the possible combinations of the full model (i.e. including two-way interactions between fixed effects) based on their Akaike's information criterion (AIC) score (akaike 1998). Analyses were performed using the R environment (R Development Core Team 2013). Unless otherwise stated, average values are reported as mean ± SE (±1 standard error).

**Table 1.** Summary table of the GLMs related to flowering time, synchrony and reproductive success of *H. balearicum* plants. Depending on each analysis and dependent variable, year, locality and flowering season are predictor factors, whereas number of flowering weeks, flowering synchrony, flower crop per plant, average receptive flowers per plant and average fruit set per plant (during off-peak flowering) are continuous predictors. The units of replication are individual plant or plant and census day. See GLM outputs in **Supporting Information File 1**.

Dependent variables			Predictor variables								Random factors	Model output
Name	Unit	Error distribution and link function	Locality	Year	Flowering season	Number of flowering weeks	Flowering synchrony	Flower crop	Avg. receptive flowers	Avg. fruit set during off-peak flowering		
Number of flowering weeks	Plant	Gaussian, log	Randa, Lluc	2001, 2002	Peak, off-peak	–	–	–	–	–	–	**Supporting Information—Table S1**
Flowering synchrony	Plant	Gaussian, log	Randa, Lluc	2001, 2002	Peak, off-peak	–	–	–	–	–	–	**Supporting Information—Table S2**
Avg. fruit set	Plant and census day	Gaussian, log	Randa, Lluc	2001, 2002	Peak, off-peak	–	–	–	–	–	–	**Supporting Information—Table S3**
Avg. fruit set during off-peak flowering	Plant and census day	Gaussian, log	Randa, Lluc	2001, 2002	–	Continuous	Continuous	Continuous	Continuous	–	–	**Supporting Information—Table S4**
Avg. fruit set during peak flowering	Plant and census day	Gaussian, log	Randa, Lluc	2001, 2002	–	Continuous	Continuous	Continuous	Continuous	Continuous	–	**Supporting Information—Table S5**

**Table 2.** Summary table of the GLMs and GLMMs related to pollinators during flowering peak of *H. balearicum*. Depending on each analysis and dependent variable, locality, year and pollinator order are predictor factors, whereas date and number receptive flowers per plant are continuous predictors. The units of replication are the visits per hour per individual plant, and individual pollinator visit (registered during each census and plant). We detected over-dispersion in the abundance of pollinators, and we thus needed to fit zero-inflated models. For analyses related to number of flowers visited and time per visit, we only considered species that had visited flowers a minimum of five times (this involved five and four species of Diptera and Hymenoptera, respectively; **see** **Supporting Information File 1**). For the analysis of time per visit, we fitted repeated measurement design that includes individual visited plant as subject random factor. See GLM and GLMM outputs in **Supporting Information File 1**.

Dependent variables			Predictor variables					Random factors	Model output
Name	Unit	Error distribution and link function	Locality	Year	Pollinator order	Date	Number of receptive flowers per plant and census day		
Pollinator richness	Visits per hour per plant	Zero-inflated Poisson, log	Randa, Lluc	2001, 2002	–	Continuous	Continuous	–	**Supporting Information—Table S6**
Pollinator abundance	Visits per hour per plant	Zero-inflated Poisson, log	Randa, Lluc	2001, 2002	–	Continuous	Continuous	–	**Supporting Information—Table S7**
Number of flowers visited	Pollinator visit	Poisson, log	Randa, Lluc	2001, 2002	Diptera, Hymeno.	–	–	–	**Supporting Information—Table S8**
Time per visit (s)	Pollinator visit	Gamma, log	Randa, Lluc	2001, 2002	Diptera, Hymeno.	–	–	Plant	**Supporting Information—Table S9**

**Table 3.** Summary table of the GLMMs related to reproductive success during flowering peak in *H. balearicum*. Depending on each analysis and dependent variable, locality and year are predictor factors, whereas date and the number of viable seeds are continuous predictors. We performed a repeated measurement design that includes individual plant as subject random factor and the individual fruit (for each plant and locality) as the unit of replication. See GLMM outputs in **Supporting Information File 1**.

Dependent variables			Predictor variables				Random factors	Model output
Name	Unit	Error distribution and link function	Locality	Year	Date	Number of viable seeds		
Fruit set	Fruit	Binomial, logit	Randa, Lluc	2000, 2001, 2002	Continuous	–	Plant	**Supporting Information—Table S10**
Seed set	Fruit	Binomial, logit	Randa, Lluc	2000, 2001, 2002	Continuous	–	Plant	**Supporting Information—Table S11**
Seed weight	Fruit	Gaussian, log	Randa, Lluc	2000, 2001, 2002	Continuous	Continuous	Plant	**Supporting Information—Table S12**

## Results

### Flowering phenology and synchrony throughout the year

Despite *H. balearicum* had receptive flowers all year round, flowering occurred mainly between early May and mid-July, which coincided with the driest period of the year at both localities (Fig. 2A and B). Rainfall decreased from early June to mid-July, and there were sporadic rainy days in the summer period until the early autumn (see Fig. 2A and B). Comparing localities, plants produced proportionally less flowers in Randa (85.4 % ± 3.4; *n* = 11; Fig. 2A) than in Lluc (95.4 % ± 1.1; *n* = 15; Fig. 2B) during the flowering peak.

The total number of flowering weeks was higher in plants at Randa than at Lluc [Fig. 2C; *t*-value = 5.50; *P* < 0.001; AIC: 695.0; df = 97; **see** **Supporting Information—Table S1**], and it was lower in 2001 (8.69 ± 1.03; both localities averaged; *t*-value = 3.69; *P* < 0.05) than in 2002 (11.61 ± 1.51; *t*-value = 3.37; *P* < 0.05). Within each flowering season (peak vs off-peak), the number of flowering weeks also varied between years and localities: it was proportionally lower in 2002 (4.74 ± 0.46; *t*-value = −2.66; *P* < 0.05) and higher in Randa during the off-peak season (21.1 ± 1.03; *t*-value = −3.85; *P* < 0.001). Flowering synchrony did not differ between flowering seasons in Randa, but did it in Lluc [*t*-value = 3.23; *P* < 0.05; AIC: −137.3; df = 91; **see** **Supporting Information—Table S2**], being greater during peak than off-peak season (Fig. 2D; *t*-value = 2.63; *P* < 0.05). Additionally, average fruit set per plant was higher during peak flowering [i.e. lower in Randa; *t*-value = 11.6; *P* < 0.001; AIC = −4.458; df = 89; **see** **Supporting Information—Table S3**], but with differences between localities: Randa set proportionally more fruits during the flowering peak, whereas Lluc during the off-peak period (Fig. 2E). A high correlation was found between flower crop and average receptive flowers per plant, and between the number of flowering weeks and the rest of variables **[see** **Supporting Information—Fig. S1****]**.

Although we expected compensation at the individual level, plants in each locality setting more fruits during the flowering off-peak did not set less fruits during the flowering peak [i.e. ‘fruit set during peak flowering’ was not selected by the best model; AIC: −4.46; df = 89; **see** **Supporting Information—Table S4**]. Furthermore, during the flowering off-peak of each locality, plants flowering for shorter periods (*t*-value = −3.62; *P* < 0.001) and less synchronic (*t*-value = −2.81; *P* < 0.05) set also more fruits than those flowering for longer periods and more synchronic; plants with larger flower crops did not affect fruit set (i.e. flower crop was not selected by the best model predicting fruit set). By contrast, plants flowering longer during the flowering peak of each locality set more fruits than plants flowering for short periods [*t*-value = 2.27; *P* < 0.05; AIC: −1.176; df = 37; **see** **Supporting Information—Table S5**].

Due to the very low fruit set of flowers during the flowering off-peak, we could not analyse either seed set or seed weight per locality for this period.

### Pollinators during the flowering peak

During the flowering peak, a similar number of pollinator species visited *H. balearicum* flowers in both localities [21 and 20 species in Randa and Lluc, respectively; **see** **Supporting Information 2—Table S13**], 48 % of the species being shared in both localities. Coleoptera was the most frequent insect order (67.6 % of total visits, both localities pooled), followed by Hymenoptera (33.7 %), Diptera (15.4 %) and Lepidoptera (0.6 %).

**Figure 3.**
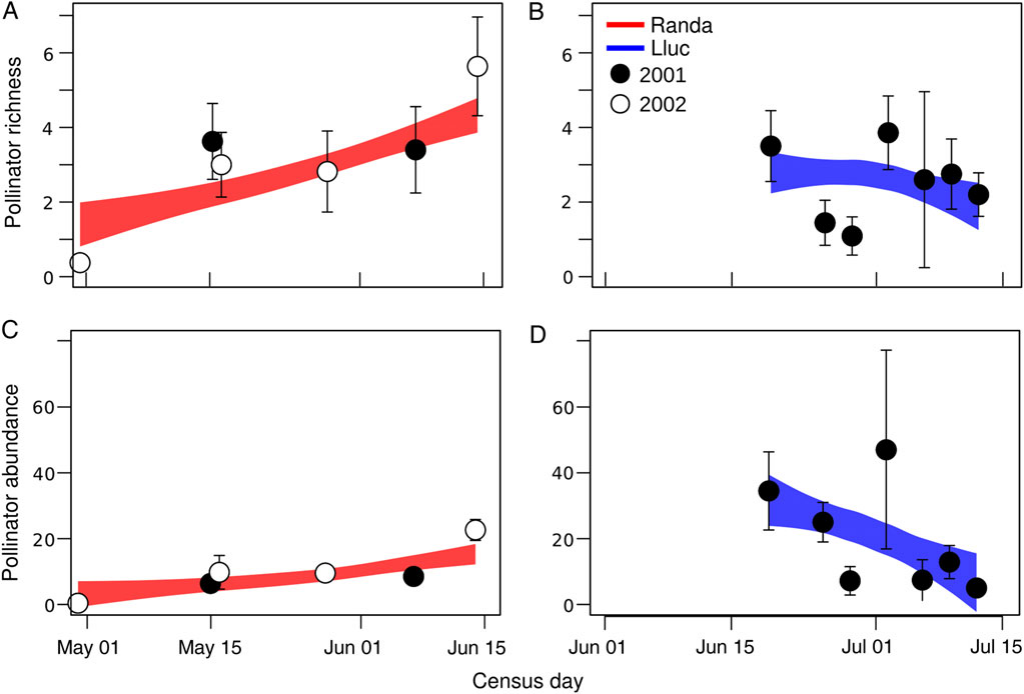
Richness and abundance of pollinators in the flowering peak for each locality of *H. balearicum*. In the left panels (A and C), we plotted values of Randa, whereas in the right panels (B and D), those of Lluc. Values (mean ± SE) for each census day are calculated at each locality, and year (circles in different colours). Predictive values (±0.95 CI) for census days of the best-fitted model (pooling years) are depicted in red (for Randa) and blue (for Lluc) areas.

Pollinator richness was higher on plants with greater flower display [*z*-value = 6.52; *P* < 0.001; AIC = 491.4, df = 7; **Supporting Information—Table S6**]. It was higher in Randa (3.26 ± 0.47 species; all census pooled) than in Lluc (2.55 ± 0.37; *z*-value = −2.2352; *P* < 0.05), and it increased along the season in Randa but not in Lluc (Fig. 3A and B; *z*-value = −1.409; *P* = 0.159). Pollinator richness was consistent between years in Randa (*z*-value = −1.734; *P* = 0.083); we did not perform this test in Lluc because it was only censused 1 year. Despite their higher species richness, pollinators were less abundant in Randa [10.2 ± 1.4 visits h^−1^; *z*-value = −19.1; *P* < 0.001; AIC = 1602.7, df = 7; **see** **Supporting Information—Table S7**] compared with Lluc (21.3 ± 4.9 visits h^−1^); in the latter, pollinator abundance was found to decrease along the season (Fig. 3D; *z*-value = −14.9; *P* < 0.001). Additionally, pollinator abundance was predicted by the same variables as pollinator richness **[see** **Supporting Information—Tables S6 and S7****]**.

Pollinators proportionally visited more flowers in plants with greater flower display [*z*-value = 2.78; *P* < 0.05; AIC = 187.5; df = 271; **Supporting Information—Table S8**], and this was found both for dipterans and hymenopterans. Beetles and ants spent long times (>>15 min) on a single flower and thus were not included in this and further analyses. Overall, pollinators in Randa visited less flowers per plant than in Lluc (*z*-value = −2.01; *P* < 0.05), although this varied across orders and localities: in Randa, hymenopterans visited more flowers (1.82 ± 0.12; *z*-value = 2.06; *P* < 0.05) than dipterans (1.05 ± 0.05), whereas no differences were observed in Lluc. Finally, visitation time of pollinators on plants was independent of the number of receptive flowers per plant, and did not differ between localities, years or across pollinator orders [i.e. effects were not significant in the best model; AIC = 2984.8; **see** **Supporting Information—Table S9**].

### Reproductive success during flowering peak

**Figure 4.**
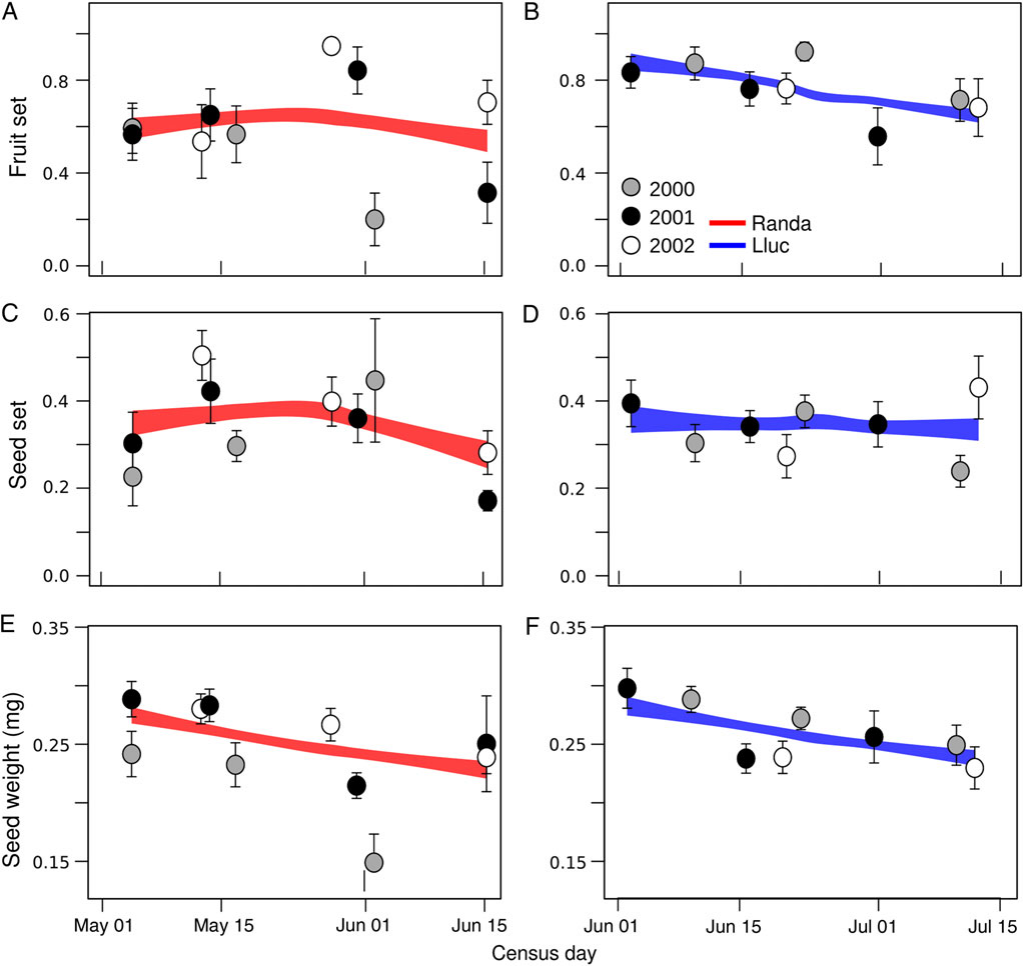
Fruit and seed set, and seed weight in the flowering peak for each locality of *H. balearicum*. Values (mean ± SE) for each census day are calculated at each locality, and year. For further details, see Fig. 3.

During the flowering peak, flowers set less fruits in Randa [57.0 % ± 3.9; *z*-value = −4.90; *P* < 0.001; AIC = 606.2; df = 546; **see** **Supporting Information—Table S10**] than in Lluc (72.9 % ± 3.2), but this pattern was not consistent along the entire season. Fruit set in Randa had a hump-shaped distribution (peaking in early June; Fig. 4A), and increased along the season (*z*-value = 2.11; *P* < 0.05); in Lluc, by contrast, it decreased along the season (i.e. highest in early June and lowest in mid-July; Fig. 4B). Seed set also differed between localities, being consistently lower in Randa (32.8 % ± 2.1; *z*-value = −6.79; *P* < 0.001) than in Lluc [34.0 % ± 1.6; AIC = 6438; df = 412; **see** **Supporting Information—Table S11**]. Seed set in Randa peaked in early June (consistently with fruit set; Fig. 4C; *z*-value = −15.1; *P* < 0.001), whereas in Lluc, it slightly increased along the season (Fig. 4D; *z*-value = 5.05; *P* < 0.001). The effects of locality, year and census day covaried with fruit and seed set **[see** **Supporting Information—Tables S10 and S11****]**.

Finally, seed weight decreased along the season [*t*-value = −5.18; *P* < 0.001; Fig. 4E and F; AIC: −887.6; df = 9; **see** **Supporting Information—Table S12**] and this was consistent in both localities and across years (i.e. ‘year’ and ‘locality’ were not selected by the best model). Seed weight was also negatively associated with the number of seeds per fruit (*t*-value = −4.83; *P* < 0.001).

## Discussion

### Flowering phenology and synchrony, and reproductive success throughout the year

In tropical and subtropical climates, flowering phenology constraints are more relaxed compared with temperate areas (Bawa *et al.* 2003), but still there is appreciable seasonality in flowering plants, mainly linked to dry seasons (Bullock 1995). In the Mediterranean basin, different flowering patterns are found: most species flower during spring and early summer (Dafni and O'Toole 1994; Proctor *et al.* 1996), whereas others do it during autumn (e.g. Picó and Retana 2000; Sánchez *et al.* 2012) or for much longer periods (e.g. Traveset 1995; Picó and Retana 2000; present study). We found that *H. balearicum* mainly flowers during the dry season (spring/summer) of the Mediterranean basin, but it also does it during autumn and winter. Flowering phenology is both under genetic control and is plastic to environment, meaning that changes in climatic conditions may trigger the expression of phenotypic responses currently hidden (Nicotra *et al*. 2010). Pollen records suggested that, during the Pliocene, Mediterranean basin was warmer and wetter than today (Suc 1984), and that subtropical climate might exerted selection in a relaxed flowering phenology of *H. balearicum.* Hence, the extended phenology observed in *H. balearicum* could be activated by changes in abiotic conditions, triggering quick and plastic flowering responses by seasonal environmental conditions under the Mediterranean climate.

As expected, during the flowering off-peak period of each locality, plants were less synchronous, produced less flowers and set less fruits. In outcrossing insect-pollinated plants, flowering during harsh seasons (i.e. winter and/or early spring season) may decrease reproduction compared with flowering during favourable periods (e.g. Elberling 2001; Anderson and Hill 2002). The lower fruit set of *H. balearicum* outside the flowering peak (which is independent of flower crop in that period) could result from two non-mutually exclusive processes: (i) the scarce insect abundance during autumn and winter in the Mediterranean basin (Dafni and O'Toole 1994) and (ii) seeds could be produced by selfing, as *H. balearicum* is self-compatible (Tébar *et al.* 2004; J. Rodríguez-Pérez, unpublished data). Our few observations of pollinators of *H. balearicum* during autumn and winter (J. Rodríguez-Pérez, pers. obs.) suggest that the former, though several mechanisms may be responsible for the low reproductive success during that period.

Comparing localities, we found that plants at Randa (the driest locality) flowered longer, were more synchronic and produced more flowers during the flowering off-peak, though they set much less (ca. six times less) fruits than plants at Lluc. It is expected that the length of favourable conditions for reproduction affects the onset and ending of the flowering season (Munguía-Rosas *et al*. 2011). One possible explanation of the differences we found in flowering phenology between localities might be related to rainfall: 2-fold in Lluc compared with Randa. In other words, a low rainfall might produce a weaker phenotypic selection on flowering phenology, as reported in other studies (Munguia-Rosas *et al*. 2011). Certainly, we would need additional data from other localities to fully understand how abiotic conditions shape the flowering phenology of *H. balearicum*. For each locality, we also found that the effect of duration of flowering outside the peak season on reproductive success was weak, as plants investing more flower resources outside the flowering peak did not set less fruits during the subsequent flowering peak. During flowering off-peak, individuals flowering for shorter periods and less synchronic proportionally set more fruits; therefore, plants with multiple reproductive events were less affected by the seasonal conditions. In short, a lower rainfall appeared to relax flowering phenology in *H. balearicum*, and to provide plants with more opportunities to reproduce outside optimum environmental conditions.

### Concordances and inconsistencies between environmental factors and reproduction during flowering peak

Richness and abundance of pollinators are important factors determining the reproductive success in most outcrossing insect-pollinated plants (Ollerton *et al*. 2011), whereas water availability likely limits the storage of resources needed for reproduction (Kudo 2006; Elzinga *et al.* 2007). In *H. balearicum*, flowers attracted a diverse array of pollinators (∼20 species at each locality), due to their conspicuousness and bowl shape. Randa proportionally received less pollinators, but (notably) hymenopterans likely promoted higher outcrossing rates (i.e. hymenopterans visited more flowers per plant and spent shorter times on a single flower) than the rest of pollinators (i.e. ants, beetles or flies spend long times in flowers, not touching stigmas; J. Rodríguez-Perez, pers. obs.). In general, plants with large flower crops attract more pollinators and have higher reproductive success than those with small flower crops (e.g. Forsyth 2003; Kudo and Harder 2005), at the expenses of higher geitonogamy levels (i.e. pollen crosses between different flowers within individual plants; De Jong et al. 1993). *Hypericum balearicum* plants can produce >500 flowers during spring and summer and their flowers are self-compatible (J. Rodríguez-Pérez, unpublished data), but its short flower lifespan and low number of receptive flowers per day (ca. six to eight flowers, on average) likely favours allogamous crosses between individuals (mainly in Randa, with lower flower visitation rates per plant). Thus, a detailed analysis of the pollinator composition and outcrossing rates within and outside the flowering peak would offer further insights into the drivers affecting flowering phenology of this species.

The reproductive success of *H. balearicum* varied along the flowering peak of each locality. In Randa, pollinator abundance might have limited reproduction as most plants flowered earlier in the season, when pollinators were not yet abundant (their richness and abundance increased along the season). On the other hand, rainfall decreased along the season in Randa, what suggests that a combination of biotic and abiotic factors related to each particular locality may limit reproduction in *H. balearicum*, notably when rainfall is more limiting. In Randa, both fruit and seed set peaked in early June, decreasing from mid-June onwards. In outcrossing self-compatible species, fruit and seed set could be considered a proxy of pollen quantity and quality, respectively, and our results may thus reflect consistency in the processes (presumably pollinator abundance and richness, respectively) affecting reproductive success. Such findings additionally indicate that when pollinators are available, it is the driest period at Randa. They also suggest that *H. balearicum* reproduction might be mostly limiting in dry years, as reported for other species (e.g. Giménez-Benavides *et al*. 2007; Sánchez *et al*. 2012).

In Lluc, by contrast, plants flowered much later, and pollinator abundance and rainfall decreased along the season (note, however, that we carried out censuses there during only one year, and thus, results on pollinator abundances and trends should be taken with caution). Here, we did neither detect differences in flower visitation rates nor visit duration between hymenopterans and dipterans, which suggest that both pollinator groups contribute similarly to reproductive success of *H. balearicum*. Despite rainfall in Lluc is much higher, water could still be limiting, and thus, the decreasing trend in fruit set along the season might result from a negative feedback between pollinator scarcity and water limitation occurring at the same time. Seed set did not vary much along the season, suggesting consistency in the processes affecting pollen quality (likely pollinator richness).

The decreasing trend in seed weight along the season was consistent in both localities, and might be related to water availability. Changes in seed weight depending on water availability during reproduction have previously been documented for other plant species in Mediterranean climates (e.g. Cavers and Steel 1984; Vaughton and Ramsey 2001). Inbreeding depression could be an additional mechanisms influencing seed size and performance (Husband and Schemske 1996; Stephenson *et al*. 2000). Flowers of *H. balearicum* are self-compatible (J. Rodríguez-Pérez, unpublished data), implying that the lower abundance of pollinators late in the season could produce proportionally more selfed seeds. Thus, it would be worth analysing the performance/establishment of seedlings derived from seeds of different sizes, and produced during the flowering peak and outside it. This coupled with the analysis of outcrossing rates could aid to better understand the evolutionary processes shaping the phenology in this species. In short, our results suggest that *H. balearicum* plants do not perform better in rainy years as pollinators may be the most limiting factor for plants flowering late in the season.

## Conclusions

Individual plants flowering for long periods have several advantages over the other members of the population related to higher reproductive success, higher outcrossing rates and more time for seed maturation (Kudo 2006; Elzinga *et al.* 2007). Insect-pollinated plants flower for longer periods than do abiotically pollinated plants, suggesting that long flowering evolves in response to phenological inconsistencies between interactions with pollinators and other biotic and abiotic factors (Elzinga *et al.* 2007; Munguia-Rosas *et al.* 2011). We found that *H. balearicum* flowers for long periods, that favourable conditions could lead to a relaxation in flowering phenology and that plants with relaxed flowering phenology likely leads to opportunities to reproduce outside the spring and summer seasons. During the flowering peak of each locality, pollinators limit reproduction in plants flowering earlier, whereas both pollinators and water resources compromise plant reproductive success in each condition (i.e. onset and ending of flowering, years and localities) related to rainfall scarcity. In seasonal climates, plants flowering for long periods are, thus, ideal model organisms to test how flowering phenology and synchrony adjust or create discrepancies with the seasonal timing of biotic and abiotic resources, and to assess how those factors generate trade-offs in plant fitness.

## Sources of Funding

This study was supported by the (former) Spanish Ministry of Science and Research (target financing project PB97-1174) and by the research grants from the Town-Hall of Ciutat de Palma (grant year 2000; Balearic Islands, Spain).

## Contributions by the Authors

J.R.-P. and A.T. designed the study. J.R.-P collected the data and analysed them. J.R.-P. led the writing with significant contributions by A.T. The two authors read and approved the final manuscript.

## Conflict of Interest Statement

None declared.

## Supporting Information

The following additional information is available in the online version of this article –

**Figure S1**. Correlogram (or correlation matrix) of number of flowering weeks (Nwk), flowering synchrony (Flsync), flower crop (Nfl), the average of receptive flowers (Flx) and average of fruit set (Frset). Upper panels depict paired plots between pair of variables, whereas lower panels show the paired (Pearson) correlation between pair of variables (the confidence intervals in brackets). We constructed correlogram using the *corrgram* library (Wright 2015).

**Table S1.** Detailed results of GLM and GLMMs comparing flowering phenology, synchrony, flower abundance and fruit set, the richness and abundance of pollinators during flowering peak and the reproductive success during flowering peak in each locality.

**Table S2.** Richness and abundance of pollinators, and number of flowers and time per each visit.

**Table S3.** Parameter estimates for the GLM analysis on the ‘Average of fruit set’ per plant by (a) Locality (Randa vs Lluc), (b) Year (2001 vs 2002) and (c) Flowering season (Peak vs Off-peak). The unit of replication was the average fruits per plant and census day (fortnightly or monthly). For abbreviations and conventions, **see** **Supporting Information—Table S1** caption. Null deviance: 12.8078 on 91 df; residual deviance: 4.7044 on 89 df; AIC: −4.4584.

**Table S4.** Parameter estimates for the GLM analysis on the ‘Fruit set during off-peak flowering’ per plant by (a) Locality (Randa vs Lluc), (b) Year (2001 vs 2002), (c) Number of flowering weeks, (d) Flowering synchrony, (e) Flower crop and (f) Number of receptive flowers per plant; we considered (a) and (b) variables as (independent) fixed effects, whereas the (c–f) variables were continuous covariates. The unit of replication was the average fruits per plant and census day (fortnightly or monthly). Response variable was fitted to a Gaussian distribution and log link function. We showed the best model (i.e. the model with the lowest AIC value from all combinations of competitive models; see Methods), and we also include in the analysis the two-way interaction between fixed variables (i.e. Locality × Year). Variables non-included in the best model were considered as non-significant. Effects with significant coefficients (*P*< 0.05) are highlighted in bold. Null deviance: 2.5013 on 40 df; residual deviance: 1.8278 on 37 df; AIC: −1.1755.

**Table S5.** Parameter estimates for the GLM analysis on the ‘Fruit set during peak flowering’ per plant by (a) Locality (Randa vs Lluc), (b) Year (2001 vs 2002), (c) Number of flowering weeks, (d) Flowering synchrony, (e) Flower crop, (f) Number of receptive flowers per plant and (g) Fruit set during off-peak flowering. For the rest of conventions, **see** **Supporting Information—Table S4**. Null deviance: 1.9071 on 40 df); residual deviance: 1.4110 on 37 df; AIC: −11.787.

**Table S6.** Parameter estimates for the GLM analysis on the ‘Pollinator richness’ per census time affected by (a) Locality (Randa vs Lluc), (b) Year (2001 vs 2002), (c) Date and (d) Number of flowers (NFl); we considered Locality and Year as (independent) fixed effects. The unit of replication was the visits per hour and individual plant. As we detected over-dispersion in response variable (i.e. phi >> 1), we fitted response variable to a zero-inflated Poisson distribution, and we thus showed estimates from the visit occurrence (zero-inflation model) and visit number (count model) using the *pscl* library (Zeileis *et al*. 2008). We showed the best model (i.e. the model with the lowest AIC value from all combinations of competitive models; see material and methods), and we also include in the analysis the two-way interaction between fixed variables (i.e. Locality × Year). Variables non-included in the best model were considered as non-significant. Effects with significant coefficients (*P* < 0.05) are highlighted in bold. Log-likelihood: −238.7 on 7 df; AIC: 491.4.

**Table S7.** Parameter estimates for the GLM analysis on the ‘Pollinators abundance’ per census time affected by (a) Locality (Randa vs Lluc), (b) Year (2001 vs 2002), (c) Date and (d) Number of receptive flowers per plant. For abbreviations and conventions, **see** **Supporting Information—Table S6** caption. Log-likelihood: −794.4 on 7 df; AIC: 1602.734.

**Table S8.** Parameter estimates for the GLM analysis on the ‘Number of flowers visited’ (flowers per visit and flowering plant) per census time affected by (a) Locality (Randa vs Lluc), (b) Year (2001 vs 2002), (c) Pollinator (Diptera vs Hymenoptera) and (d) Number of flowers; we considered Locality, Year and Flower visitor as (independent) fixed factors. The unit of replication was the individual pollinator visit (registered during each census and individual plant). Response variable was fitted to a Poisson distribution and log link function. We showed the best model (i.e. the model with the lowest AIC value from all combinations of competitive models; see material and methods), and we also include in the analysis the two-way interaction between fixed variables (i.e. Locality × Year). Variables non-included in the best model were considered as non-significant. Effects with significant coefficients (*P* < 0.05) are highlighted in bold. Residual deviance: 175.5 on 271 df; AIC: 187.5.

**Table S9.** Parameter estimates for the GLM analysis on the ‘Time per visit’ (in seconds) in each individual plant and census affected by (a) Locality (Randa vs Lluc), (b) Year (2001 vs 2002), (c) Pollinator (Diptera vs Hymenoptera) and (d) Number of flowers. Response variable was fitted to a gamma distribution and log link function. The unit of replication was the individual pollinator visit (registered during each census and plant). We fitted mixed models with individual visited plant as within-group random factor using the *glmmML* library (Broström 2013). For abbreviations and conventions, **see** **Supporting Information—Table S8** caption. Residual random effects: 9872; AIC: 2984.766.

**Table S10.** Parameter estimates for the GLM analysis on the ‘Fruit set’ (flowers setting fruits with at least one viable seed) affected by (a) Locality (Randa vs Lluc), (b) Year (2000, 2001 and 2002) and (c) Date (during the flowering peak); we considered Locality and Year as (independent) fixed effects. We performed a repeated measurement design that includes individual plant as subject random factor and the individual fruit (for each plant and locality) as the unit of replication. We fitted mixed models with individual plant as within-group random factor using the *glmmML* library (Broström 2013). Response variable was fitted to a binomial distribution and logit link function. We showed the best model (i.e. the model with the lowest AIC value from all combinations of competitive models; see material and methods), and we also include in the analysis the two-way interaction between fixed variables (i.e. Locality × Year). Variables non-included in the best model were considered as non-significant. Effects with significant coefficients (*P* < 0.05) are highlighted in bold. Residual deviance 636.2 on 546 df; AIC: 606.2.

**Table S11.** Parameter estimates for the GLM analysis on the ‘Seed set’ (ovules setting viable seeds per fruit) affected by (a) Locality (Randa vs Lluc), (b) Year (2000, 2001 and 2002) and (c) Date (during the flowering peak); we considered Locality and Year as (independent) fixed effects. For abbreviations and conventions, **see** **Supporting Information—Table S8** caption. Residual deviance 6408 on 412 df; AIC: 6438.

**Table S12.** Parameter estimates for the GLM analysis on the ‘Seed weight’ (weight of viable seeds in mg divided by the number seeds per fruit) affected by (a) Locality (Randa vs Lluc), (b) Year (2000, 2001 and 2002), (c) Date (during the flowering peak) and (d) number of seeds (NSD); we considered Locality and Year as (independent) fixed effects. We fitted mixed models with individual plant as within-group random factor using the *nlme* library (Pinheiro *et al*. 2014). Response variable was fitted to a Gaussian distribution and log link function. For abbreviations and conventions, **see** **Supporting Information—Table S8** caption. Log-likelihood: 448.792 on 9 df; AIC: −887.585.

**Table S13.** Pollinator richness, flower visit rate (visits per hour, flowering plant and locality), number of visited flowers (flowers per visit and flowering plant, localities pooled) and time per visit (in seconds per flowering plant, localities pooled). We showed average values (±1 SE) and number of observation per species (in brackets). For Coleoptera and Formicidae, we only showed visit presence since most visits lasted longer than census time (15 min), visiting only one flower (pers. obs.).

Additional Information
