# Supplementary material for: Effects of flowering phenology and synchrony on the reproductive success of a long-flowering shrub
Source: AoB Plants. 2016 Feb 2;8:plw007. doi: 10.1093/aobpla/plw007 (PMC4793561; doi:10.1093/aobpla/plw007)
Supplement: Additional Information [file supp_8_plw007_index.html]

Effects of flowering phenology and synchrony on the reproductive success of a long-flowering shrub — Effects of flowering phenology and synchrony on the reproductive success of a long-flowering shrub — Additional Information 

# Effects of flowering phenology and synchrony on the reproductive success of a long-flowering shrub

## Additional Information

Additional Information

- Supplementary File 1 - doc file
- Supplementary File 2 - doc file
