# Supplementary material for: Effects of flowering phenology and synchrony on the reproductive success of a long-flowering shrub
Source: AoB Plants. 2016 Feb 2;8:plw007. doi: 10.1093/aobpla/plw007 (PMC4793561; doi:10.1093/aobpla/plw007)
Supplement: Additional Information [file supp_plw007_plw007supp_file1.doc]

**Supporting Information File 1** – Correlogram and detailed results of Generalized Linear Models (GLM) and Generalized Linear Mixed Models (GLMMs) comparing number of flowering weeks, flowering synchrony and average of fruit set (Figure A1; Tables A1 to A5), the richness and abundance of pollinators during flowering peak (Tables A6 to A9), and the reproductive success during flowering peak (Tables A10 to A12) in each locality.

**Figure A1**. Correlogram (or correlation matrix) of number of flowering weeks (Nwk), flowering syncrhony (Flsync), flower crop (Nfl), the average of receptive flowers (Flx), and average of fruit set (Frset). Upper panels depict paired plots between pair of variables, whereas lower panels show the paired (pearson) correlation between pair of variables (the confidence intervals in brackets). We constructed correlogram using the *corrgram* library (Wright 2015).


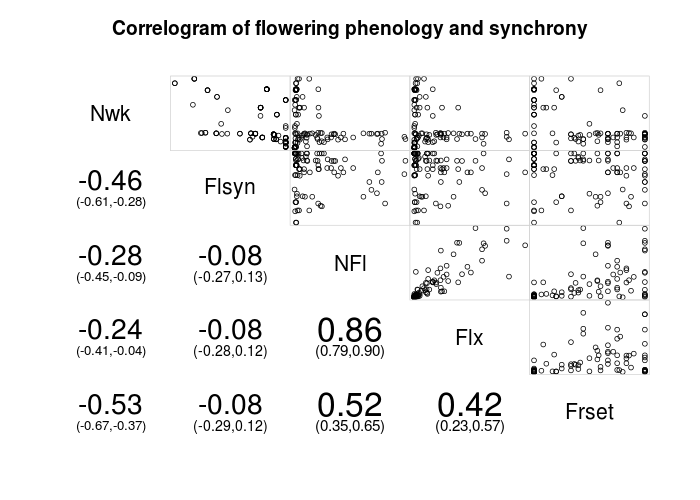


**Table A1.** Parameter estimates for the GLM analysis on the “Number of flowering weeks” (response variable) per plant by (a) Locality (Randa vs Lluc), (b) Year (2001 vs 2002) and c) Flowering season (Peak vs. Off-peak). The unit of replication was individual plant. Flowering season (Peak vs. Off-peak); we considered aforementioned variables as (independent) fixed effects. Response variable was fitted to a gaussian distribution and log link function. We showed the best model (i.e. the model with the lowest AIC value from all combinations of competitive models; see material and methods), and we also include in the analysis the two-way interaction between fixed variables (i.e. Locality x Year). Variables non-included in the best model were considered as non-significant. Effects with significant coefficients (*p*<0.05) are highlighted in bold. Null deviance: 8885.2 on 102 degrees of freedom (d.f.); Residual deviance: 4483.6 on 97 d.f.; AIC: 695.0.

Fixed effects: Estimate Std. Error t value Pr(>|t|)

**(Intercept) 7.377 1.559 4.732 7.55e-06**

**Loc.Randa 10.487 1.908 5.495 3.15e-07**

**Year.2002 6.352 1.886 3.368 0.001085**

Flw.Peak -1.840 2.207 -0.834 0.406407

**Loc.Randa:Flw.Peak -10.415 2.709 -3.845 0.000216**

**Year.2002:Flw.Peak -7.127 2.680 -2.659 0.009162**

**Table A2.** Parameter estimates for the GLM analysis on the “Flowering synchrony” per plant by (a) Locality (Randa vs Lluc), (b) Year (2001 vs 2002) and (c) Flowering season (Peak vs. Off-peak). For abbreviations and conventions, see Table A1 caption. Null deviance: 1.3387 on 94 d.f.; Residual deviance: 1.1800 on 91 d.f.; AIC: -137.3.

Fixed effects: Estimate Std. Error t value Pr(>|t|)

**(Intercept) 0.81447 0.02428 33.547 < 2e-16**

**Loc.Randa 0.11099 0.03433 3.233 0.00171**

**Flw.Peak 0.08463 0.03220 2.629 0.01006**

**Loc.Randa:Flw.Peak -0.10637 0.04707 -2.260 0.02621**

**Table A3.** Parameter estimates for the GLM analysis on the “Average of fruit set” per plant by (a) Locality (Randa vs Lluc), (b) Year (2001 vs 2002) and (c) Flowering season (Peak vs. Off-peak). The unit of replication was the average fruits per plant and census day (fortnightly or monthly). For abbreviations and conventions, see Table A1 caption. Null deviance: 12.8078 on 91 d.f.; Residual deviance: 4.7044 on 89 d.f.; AIC: -4.4584.

Fixed effects: Estimate Std. Error t value Pr(>|t|)

**(Intercept) 0.19573 0.04353 4.497 2.07e-05**

**Loc.Randa -0.12311 0.04815 -2.557 0.0123**

**Flw.Peak 0.57267 0.04829 11.859 < 2e-16**

**Table A4.** Parameter estimates for the GLM analysis on the “Fruit set during off-peak flowering” per plant by (a) Locality (Randa vs Lluc), (b) Year (2001 vs 2002), (c) Number of flowering weeks, (d) Flowering synchrony, (e) Flower crop, and (f) Number of receptive flowers per plant; we considered a) and b) variables as (independent) fixed effects, whereas the c-f variables were continuous covariates. The unit of replication was the average fruits per plant and census day (fortnightly or monthly). Response variable was fitted to a gaussian distribution and log link function. We showed the best model (i.e. the model with the lowest AIC value from all combinations of competitive models; see material and methods), and we also include in the analysis the two-way interaction between fixed variables (i.e. Locality x Year). Variables non-included in the best model were considered as non-significant. Effects with significant coefficients (*p*<0.05) are highlighted in bold. Null deviance: 2.5013 on 40 degrees of freedom (d.f.); Residual deviance: 1.8278 on 37 d.f.; AIC: -1.1755.

Fixed effects: Estimate Std. Error t value Pr(>|t|)

(Intercept) -2.654e+02 1.587e+02 -1.672 0.10294

Year 1.333e-01 7.938e-02 1.680 0.10148

**Flsyn -1.173e+00 3.242e-01 -3.619 0.00088**

**Nwk -1.438e-02 5.117e-03 -2.810 0.00787**

**Table A5.** Parameter estimates for the GLM analysis on the “Fruit set during peak flowering” per plant by (a) Locality (Randa vs Lluc), (b) Year (2001 vs 2002), (c) Number of flowering weeks, (d) Flowering synchrony, (e) Flower crop, (f) Number of receptive flowers per plant and g) Fruit set during off-peak flowering. For the rest of conventions, see Table A4. Null deviance: 1.9071 on 40 degrees of freedom (d.f.); Residual deviance: 1.4110 on 37 d.f.; AIC: -11.787.

Fixed effects: Estimate Std. Error t value Pr(>|t|)

(Intercept) -2.447e+02 1.261e+02 -1.941 0.0599

Year 1.225e-01 6.298e-02 1.945 0.0594

NFl 3.115e-04 1.651e-04 1.887 0.0671

**Nwk 3.381e-02 1.492e-02 2.267 0.0293**

**Table A6.** Parameter estimates for the GLM analysis on the “Pollinator richness” per census time affected by (a) Locality (Randa vs Lluc), (b) Year (2001 vs 2002), (c) Date and (d) Number of flowers (NFl); we considered Locality and Year as (independent) fixed effects. The unit of replication was the visits per hour and individual plant. As we detected over-dispersion in response variable (i.e. phi>>1), we fitted response variable to a zero-inflated poisson distribution, and we thus showed estimates from the visit occurrence (zero-inflation model) and visit number (count model) using the *pscl* library (Zeileis et al. 2008). We showed the best model (i.e. the model with the lowest AIC value from all combinations of competitive models; see material and methods), and we also include in the analysis the two-way interaction between fixed variables (i.e. Locality x Year). Variables non-included in the best model were considered as non-significant. Effects with significant coefficients (*p*<0.05) are highlighted in bold. Log-Likelihood: -238.7 on 7 degrees of freedom (d.f.); AIC: 491.4.

Count model coefficients (poisson with log link):

Fixed effects: Estimate Std. Error z value Pr(>|z|)

**(Intercept) 1.415717 0.344122 4.114 3.89e-05**

**Loc.Randa -0.963638 0.431529 -2.233 0.02554**

Year.2002 -0.290102 0.167288 -1.734 0.08289

**NFl 0.039900 0.006122 6.517 7.15e-11**

Loc.Lluc:Date -0.015793 0.011205 -1.409 0.15870

Zero-inflation model coefficients (binomial with logit link):

Estimate Std. Error z value Pr(>|z|)

**(Intercept) -0.9682 0.2336 -4.145 3.4e-05**

**Table A7.** Parameter estimates for the GLM analysis on the “Pollinators abundance” per census time affected by (a) Locality (Randa vs Lluc), (b) Year (2001 vs 2002), (c) Date and (d) Number of receptive flowers per plant. For abbreviations and conventions, see Table A6 caption. Log-Likelihood: -794.4 on 7 d.f.; AIC: 1602.734.

Count model coefficients (poisson with log link):

Fixed effects: Estimate Std. Error z value Pr(>|z|)

**(Intercept) 4.515922 0.118126 38.230 < 2e-16**

**Loc.Randa -3.874141 0.202545 -19.127 < 2e-16**

Year.2002 0.148892 0.102866 1.447 0.148

**NFl 0.062802 0.002309 27.198 < 2e-16**

**Loc.Lluc:Date -0.065952 0.004431 -14.886 < 2e-16**

**Loc.Randa:Date 0.021348 0.003091 6.906 4.99e-12**

Zero-inflation model coefficients (binomial with logit link):

Estimate Std. Error z value Pr(>|z|)

**(Intercept) -1.6536 0.2606 -6.347 2.2e-10**

**Table A8.** Parameter estimates for the GLM analysis on the “Number of flowers visited ” (flowers per visit and flowering plant) per census time affected by (a) Locality (Randa vs Lluc), (b) Year (2001 vs 2002), (c) Pollinator (Diptera vs Hymenoptera) and (d) Number of flowers; we considered Locality, Year and Flower visitor as (independent) fixed factors. The unit of replication was the individual pollinator visit (registered during each census and individual plant). Response variable was fitted to a poisson distribution and log link function. We showed the best model (i.e. the model with the lowest AIC value from all combinations of competitive models; see material and methods), and we also include in the analysis the two-way interaction between fixed variables (i.e. Locality x Year). Variables non-included in the best model were considered as non-significant. Effects with significant coefficients (*p*<0.05) are highlighted in bold. Residual deviance: 175.5 on 271 degrees of freedom (d.f.); AIC: 187.5.

Fixed effects: coef se(coef) z-value Pr(>|z|)

**(Intercept) 0.31522 0.096291 3.2736 0.00106**

**Loc.Randa -0.45807 0.227514 -2.0134 0.04410**

Pol.Hym 0.05758 0.138112 0.4169 0.67700

**NFl 0.01419 0.005102 2.7810 0.00542**

**Loc.Randa:Pol.Hym 0.54335 0.263948 2.0585 0.03950**

**Table A9.** Parameter estimates for the GLM analysis on the “Time per visit” (in seconds) in each individual plant and census affected by (a) Locality (Randa vs Lluc), (b) Year (2001 vs 2002), (c) Pollinator (Diptera vs Hymenoptera) and (d) Number of flowers. Response variable was fitted to a gamma distribution and log link function. The unit of replication was the individual pollinator visit (registered during each census and plant). We fitted mixed models with individual visited plant as within-group random factor using the *glmmML* library (Broström 2013). For abbreviations and conventions, see Table A8 caption. Residual random effects: 9872; AIC: 2984.766.

Random effects:

Groups Name Variance Std.Dev.

Ind (Intercept) 0 0.00

Residual 9872 99.36

Number of obs: 251, groups: Ind, 20

Fixed effects: Estimate Std.Error df t-value Pr(>|t|)

**(Intercept) 53.4425 12.3494 243.9900 4.328 2.2e-05**

Loc.Randa 49.2083 32.9993 244.0000 1.491 0.1372

Year.2002 -68.5556 45.8049 244.0000 -1.497 0.1358

Pol.Hym 6.3651 18.2981 244.0000 0.348 0.7283

NFl -0.1964 0.7437 243.9900 -0.264 0.7920

Loc.Randa:Pol.Hym -69.6017 40.6071 244.0000 -1.714 0.0878

Year.2002:Pol.Hym 94.9688 50.8082 244.0000 1.869 0.0628

**Table A10.** Parameter estimates for the GLM analysis on the “Fruit set” (flowers setting fruits with at least one viable seed) affected by (a) Locality (Randa vs Lluc), (b) Year (2000, 2001 and 2002), and (c) Date (during the flowering peak); we considered Locality and Year as (independent) fixed effects. We performed a repeated measurement design which includes individual plant as subject random factor and the individual fruit (for each plant and locality) as the unit of replication. We fitted mixed models with individual plant as within-group random factor using the *glmmML* library (Broström 2013). Response variable was fitted to a binomial distribution and logit link function. We showed the best model (i.e. the model with the lowest AIC value from all combinations of competitive models; see material and methods), and we also include in the analysis the two-way interaction between fixed variables (i.e. Locality x Year). Variables non-included in the best model were considered as non-significant. Effects with significant coefficients (*p*<0.05) are highlighted in bold. Residual deviance 636.2 on 546 d.f; AIC: 606.2.

Fixed effects: coef se(coef) z Pr(>|z|)

**(Intercept) 4.3378486 1.016488 4.2675 1.98e-05**

**Loc.Randa -4.3432435 0.886640 -4.8985 9.65e-07**

**Year.2001 -2.7629513 0.938775 -2.9431 3.25e-03**

**Year.2002 -5.6788322 2.452505 -2.3155 2.06e-02**

FlwDate -0.1229639 0.092212 -1.3335 1.82e-01

I(FlwDate^2) 0.0007445 0.001958 0.3802 7.04e-01

**Loc.Randa:Year.2001 2.6804823 0.555821 4.8226 1.42e-06**

**Loc.Randa:Year.2002 3.2110883 0.698603 4.5964 4.30e-06**

**Loc.Randa:Date 0.1530282 0.072483 2.1112 3.48e-02**

Loc.Randa:I(Date^2) -0.0025074 0.001558 -1.6092 1.08e-01

Year.2001:Date 0.1061835 0.090383 1.1748 2.40e-01

**Year.2002:Date 0.3322448 0.198622 1.6727 9.44e-02**

Year.2001:I(Date^2) -0.0016009 0.002096 -0.7638 4.45e-01

Year.2002:I(Date^2) -0.0045824 0.003637 -1.2600 2.08e-01

**Table A11.** Parameter estimates for the GLM analysis on the “Seed set” (ovules setting viable seeds per fruit) affected by (a) Locality (Randa vs Lluc), (b) Year (2000, 2001 and 2002), and (c) Date (during the flowering peak); we considered Locality and Year as (independent) fixed effects. For abbreviations and conventions, see Table A8 caption. Residual deviance 6408 on 412 d.f; AIC: 6438.

Fixed effects: coef se(coef) z Pr(>|z|)

**(Intercept) -1.3209093 0.0768598 -17.186 0.00e+00**

**Loc.Randa -0.4297448 0.0632639 -6.793 1.10e-11**

**Year.2001 0.4115633 0.0650393 6.328 2.48e-10**

**Year.2002 0.4598569 0.1431932 3.211 1.32e-03**

**Loc.Randa:Year.2001 0.1273166 0.0442694 2.876 4.03e-03**

**Loc.Randa:Year.2002 0.4606823 0.0524390 8.785 0.00e+00**

**Loc.Lluc:Date 0.0309979 0.0061430 5.046 4.51e-07**

**Loc.Randa:Date 0.0830574 0.0066883 12.418 0.00e+00**

**Loc.Lluc:I(Date^2) -0.0008387 0.0001358 -6.177 6.52e-10**

**Loc.Randa:I(Date^2) -0.0023511 0.0001554 -15.127 0.00e+00**

**Year.2001:Date -0.0386131 0.0069782 -5.533 3.14e-08**

**Year.2002:Date -0.0797535 0.0117982 -6.760 1.38e-11**

**Year.2001:I(Date^2) 0.0010970 0.0001707 6.425 1.32e-10**

**Year.2002:I(Date^2) 0.0020612 0.0002253 9.147 0.00e+00**

**Table A12.** Parameter estimates for the GLM analysis on the “Seed weight” (weight of viable seeds in mg divided by the number seeds per fruit) affected by (a) Locality (Randa vs Lluc), (b) Year (2000, 2001 and 2002), (c) Date (during the flowering peak) and (d) number of seeds (NSD); we considered Locality and Year as (independent) fixed effects. We fitted mixed models with individual plant as within-group random factor using the *nlme* library (Pinheiro et al. 2014). Response variable was fitted to a gaussian distribution and log link function. For abbreviations and conventions, see Table A8 caption. Log-Likelihood: 448.792 on 9 d.f.; AIC: -887.585.

Random effects: Formula: ~1 | Ind

(Intercept) Residual

StdDev: 0.02154352 0.06911353

Fixed effects: Value Std.Error DF t-value p-value

**(Intercept) 0.31233872 0.010617429 363 29.417546 0**

**Date -0.00145481 0.000281087 363 -5.175663 0**

**NSD -0.00042691 0.000088371 363 -4.830868 0**

**Literature Cited**

Broström G. 2013. glmmML: Generalized linear models with clustering. R package version 1.0. <http://CRAN.R-project.org/package=glmmML>

Pinheiro J, Bates D, DebRoy S, Sarkar D and R Core Team. 2014. _nlme: Linear and Nonlinear Mixed Effects Models. R package version 3.1-117, <URL: <http://CRAN.R-project.org/package=nlme>>.

Wright K. 2015. corrgram: Plot a Correlogram. R package version 1.8. http://CRAN.R-project.org/package=corrgram

Zeileis A, Kleiber C, Jackman S. 2008. Regression Models for Count Data in R. *Journal of Statistical Software* 27(8): 1-25.
