# Supplementary material for: Effects of flowering phenology and synchrony on the reproductive success of a long-flowering shrub
Source: AoB Plants. 2016 Feb 2;8:plw007. doi: 10.1093/aobpla/plw007 (PMC4793561; doi:10.1093/aobpla/plw007)
Supplement: Additional Information [file supp_plw007_plw007supp_file2.doc]

**Supporting Information File 2** – Richness and abundance of pollinators, and number of flowers and time per each visit.

**Table B1.** Pollinator richness, flower visit rate (visits per hour, flowering plant and locality), number of visited flowers (flowers per visit and flowering plant, localities pooled), and time per visit (in seconds per flowering plant, localities pooled). We showed average values (± 1 SE) and number of observation per species (in brackets). For Coleoptera and Formicidae, we only showed visit presence since most visits lasted longer than census time (15 minutes), visiting only one flower (pers. obs.).

| **Order** | **Family** | Species | **Flower visit**  **in Randa** | **Flower visit**  **in Lluc** | **Number of visited flowers** | **Time per visit (s)** |
| --- | --- | --- | --- | --- | --- | --- |
| Diptera | Syrphidae | Dasysyrphus albostriatus | 0.10 (3) | - | 1.3 ± 0.3 (3) | 19.2 ± 10.5 (3) |
|  | Syrphidae | *Chrysotoxum intermedium* | 0.17 (5) | 0.38(9) | 1.6 ± 0.2 | 68.8 ± 25.5 (14) |
|  | Syrphidae | *Eristalis tenax* | 0.10 (3) | 0.09 (2) | 1.0 ± 0.0 (4) | 14.3 ± 0.9 (3) |
|  | Syrphidae | *Sphaerophoria scripta* | 0.07 (2) | 0.26 (6) | 1.1 ± 0.1 (8) | 55.7 ± 28.9 (8) |
|  | Syrphidae | *Eristalinus taeniops* | - | 0.09 (2) | 2.0 ± 1.0 (2) | 18.3 ± 8.1 (2) |
|  | Calliphoridae | *Stomorhina lunata* | 0.27 (8) | 1.15 (27) | 1.1 ± 0.1 (22) | 132.3 ± 40.9 (22) |
|  | Bombyliidae | *Hemipenthes morio* | 0.24 (7) | 1.70 (40) | 1.5 ± 0.2 (43) | 31.4 ± 5.7 (38) |
|  | Bombyliidae | *Villa hottentotta* | 0.44 (13) | 1.53 (36) | 1.9 ± 0.5 (29) | 26.3 ± 8.3 (24) |
|  |  |  |  |  |  |  |
| Coleoptera | Oedemeridae | Oedemera sp.1 | 0.64 (19) | 0.04 (1) | - | - |
|  | Buprestidae | Buprestidae sp.1 | 0.97 (29) | 0.34 (8) | - | - |
|  | Buprestidae | Buprestidae sp.2 | - | 0.17 (4) | - | - |
|  | Cantharidae | Cantharidae sp.1 | 2.39 (71) | 22.0 (516) | - | - |
|  | Mordellidae | *Anaspis* cf. *regimbarti* | 1.45 (43) | 0.04 (1) | - | - |
|  |  | Coleoptera sp.1 | 0.47 (14) | - | - | - |
|  |  | Coleoptera sp.2 | 0.24 (7) | - | - | - |
|  |  | Coleoptera sp.3 | - | 0.04 (1) | - | - |
|  |  |  |  |  |  |  |
| Hymenoptera | Apidae | Apis mellifera | 0.07 (2) | 0.04 (1) | 1.0 ± 0.0 (3) | 2.2 ± 1.1 (3) |
|  | Apidae | *Bombus terrestris* | 0.07 (2) | - | 1.5 ± 0.5 (2) | 9.5 ± 7.9 (2) |
|  | Halictidae | *Lasioglossum* sp. | 2.76 (82) | 2.21 (52) | 2.98 ± 0.13 (90) | 73.2 ± 11.0 (97) |
|  | Halictidae | *Halictus fulvipes* | 0.27 (8) | - | 1.88 ± 0.4 (8) | 14.2 ± 4.3 (8) |
|  | Halictidae | *Halictus gemmeus* | - | 0.26 (6) | 3.0 ± 0.8 (6) | 24.2 ± 9.5 (6) |
|  | Megachilidae | *Osmia caerulescens* | 0.13 (4) | - | 2.5 ± 0.6 (4) | 7.4 ± 1.7 (4) |
|  | Megachilidae | *Megachile* sp. | 0.50 (15) | - | 2.2 ± 0.3 (15) | 10.1 ± 1.5 (14) |
|  | Formicidae | *Formicidae* spp. | 0.20 (6) | 7.57 (178) | - | - |
| - |  |  |  |  |  |  |
| Lepidoptera | Pieridae | *Pieris rapae* | 0.03 (1) | 0.04 (1) | 1.0 ± 0.0 (2) | 0.9 ± 0.7 (2) |
|  | Satyridae | *Pararge aegeria* | - | 0.13 (3) | 1.7 ± 0.7 (3) | 50.1 ± 39.4 (3) |
|  | Sphingidae | *Macroglossum stellatarum* | - | 0.04 (1) | 1.0 (1) | 0.5 (1) |
